# Association between air pollution in the 2015 winter in South Korea and population size, car emissions, industrial activity, and fossil-fuel power plants: an ecological study

**DOI:** 10.1186/s40557-018-0273-5

**Published:** 2018-10-05

**Authors:** Hyeran Choi, Jun-Pyo Myong

**Affiliations:** Department of Occupational & Environmental Medicine, Seoul St. Mary’s Hospital, College of Medicine, The Catholic University of Korea, Seoul, Republic of Korea

**Keywords:** PM_10_, CO, Population density, Number of car registration, Car accident count, Industrial power usage, Fossil-fuel power plant

## Abstract

**Background:**

Compared to 10 years ago, the ambient particulate matter 10 (PM_10_) and carbon monoxide (CO) levels in South Korea have decreased. However, compared to many other OECD countries, these levels are still too high. Concentration of air pollutants such as PM_10_ is especially higher during winter than during summer. The first step to rationally solving the air pollution problem in Korea is to identify the key air pollution sources during each season. This ecological study was performed to assess the association between the number of days the accepted PM_10_ and CO thresholds were exceeded and the concentration of potential emission sources in winter season 2015.

**Methods:**

An emission inventory of the PM_10_ and CO emissions in the 232 administrative South Korean districts in January, 2015, and February, 2015 and December, 2015, and the population density, number of car registrations, number of car accidents, industrial power usage, and presence of a fossil-fuel power plant in each district was established on the basis of official web-page data from the government. For all emission source variables except power plants, the administrative districts were grouped into quartiles. Districts were also divided according to whether a power plant was present or not. Negative binomial regression was performed to assess the associations between the PM_10_ and CO air pollution (defined as ≥100 g/m^3^ and ≥ 9 ppm, respectively) and the concentration of each emission source.

**Results:**

Compared to the districts with the lowest population density, the districts with the third highest population density associated most strongly with air pollution. This was also observed for industrial power usage. Car accident number and car registration numbers showed a linear relationship with air pollution. Districts with power plants were significantly more likely to have air pollution than districts that lacked a plant.

**Conclusions:**

Greater car numbers, industrial activity, and population density, and the presence of fossil-fuel plants associated with air pollution in the 2015 winter in South Korea. These data highlight the contaminant sources that could be targeted by interventions that aim to reduce air pollution, decrease the incidence of exposure, and limit the impact of pollution on human health.

**Electronic supplementary material:**

The online version of this article (10.1186/s40557-018-0273-5) contains supplementary material, which is available to authorized users.

## Background

Environmental white papers published in 2017 by the Ministry of Environment of South Korea state that while the atmospheric concentrations of nitrogen oxide (NO_2_) and ozone (O_3_) have not changed in the past 10 years, the levels of sulfur oxides (SO_2_) and particulate matter 10 (PM_10_) have dropped. In particular, the PM_10_ concentration has decreased markedly. However, it is still relatively high compared to the levels in many of the other OECD countries [1]. This is significant because many recent studies show that high particulate matter concentrations are a major public health issue [2–11]. These findings have intensified efforts to identify the sources of particulate matter in South Korea and other countries.

Particulate matter can be divided into two types: one is emitted by directly from a source while the other is the result of a chemical reaction. The primary particulate matter is mainly emitted by diesel cars and factory chimneys while the secondary particulate matter is the result of chemical reactions with the SO_2_ and NO_2_ that is emitted by automobiles or plants [12]. Several studies in Germany, South Korea, India, and China show that PM_10_ is mostly due to secondary sulfate reactions, secondary nitrogen reactions, and diesel and gasoline cars. Other common sources are construction, road dust, soil, other combustion reactions, incineration, and industry-related activity [13–16].

According to a recent report on seasonal variation of air pollution level in Poland, the concentrations of PM_10_, carbon monoxide (CO), NO_2_, and SO_2_ rise in winter because of the increase in heat consumption. In particular, the PM_10_ concentrations are highest in winter, followed by autumn, spring, and summer. O_3_ concentrations tend to increase during the summer because of increased frequencies of the photochemical reaction [17]. A study on the long-term trends of particulate matter in the air in a Seoul urban area from 2004 to 2013 showed that the average concentration of PM_2.5_ was highest in the winter, while PM_10_ was also higher in winter than during summer. Both PM_10_ and PM_2.5_ had the lowest concentration during summer [18]. This seasonal variation and increased PM in winter might be attributed to strong fuel consumption and temperature inversion associated with the winter season [19, 20]. The PM_10_ levels correlate with various air pollution sources such as industrial activities, on road, and off road. However, a detailed association between air pollution sources and PM_10_ as well as the nationwide results were not shown.

The first step to solve the air pollution problem in Korea is to identify the key sources of pollutants. The purpose of this ecological study was to estimate the association between the number of days the PM_10_ and CO levels exceeded the exposure limits in the winter of 2015 and the concentration of several potential pollution sources.

## Methods

### Generation of an emission inventory for South Korea in the winter of 2015

South Korea has 232 administrative districts that are termed Si-goon-gu. The total air pollutants that were emitted in each Si-goon-gu from several potential pollutant sources in the 2015 winter (January, 2015, February, 2015 and December, 2015) were estimated, as follows.

#### Population density

The population density per unit area (persons/km^2^) in each Si-goon-gu in December 2015 was calculated on the basis of data provided by the Ministry of Land, Infrastructure, and Transport [21] [22]. To assess the association between population density and air pollution, the 232 Si-goon-gu were divided into quartile groups on the basis of the population densities: in first quartile (Q1), secondary quartile (Q2), third quartile (Q3), and fourth quartile (Q4), the population density was < 98.27, 98.27–459.19, 459.19–6329.12, and ≥ 6329.12 persons/km^2^, respectively.

#### Number of car registrations

The total number of registered motor vehicles in 2015 in each administrative district was obtained from the Ministry of Land, Infrastructure, and Transport [23]. Since commercial trucks may not actually be located in the area of registration, we excluded vans, trucks, and other car types that are likely to have variable locations; only the car registration data were used. The data were expressed as number of car registrations/1000. The 232 Si-goon-gu were divided into quartile groups on the basis of the car registrations/1000: in Q1, Q2, Q3, and Q4, the number of car registrations/1000 was < 16.43, 16.43–48.90, 48.90–98.36, and ≥ 98.36, respectively.

#### Number of car accidents

Park found that the number of automobile accidents increases in proportion to the traffic density [24]. Therefore, the number of car accidents is a valuable surrogate marker of mobile sources of air pollution. The number of car accidents in the winter of 2015 was obtained from the Road Traffic Authority [25]. The number of car accidents in each administrative district was expressed simply as counts (i.e., not in relation to unit area). The 232 Si-goon-gu were divided into quartile groups on the basis of the car accident counts: in Q1, Q2, Q3, and Q4, the number of car accidents were < 213, 213–728, 729–1520, and ≥ 1521, respectively.

#### Industrial power usage

The Korea Electric Power Corporation (KEPCO) is the exclusive provider of electricity power to the nation [26]. Data from the KEPCO were used to estimate the industrial power usage in each district. The data from each administrative district were expressed as kilowatt-hour (kwh)/km^2^. The 232 Si-goon-gu were divided into quartile groups on the basis of their industrial power usage: in Q1, Q2, Q3, and Q4, the industrial power usage was < 300,926.7, 300,926.7–1,588,265.6, 300,926.7–8,306,667.3, and ≥ 8,306,667.4 kwh/km^2^, respectively.

#### Generation of electricity

The amount of power that was generated from fossil fuels was estimated on the basis of detailed statistics on the power plants from the KEPCO and nationwide statistics reported by the Korean government on the “public data portal” web-page (http://epsis.kpx.or.kr/epsisnew/selectEkfaFclDtlChart.do?menuId=020104) [27]. The KEPCO also provided, on request, data on how much electricity was generated by each power plant. These data were used to determine how much power (kwh) was used in the 2015 winter and the location of each plant. Since only 90 out of 232 administrative districts had a power plant, the generation of electricity was expressed as a dichotomous variable (Yes/No).

### Assessment of CO and PM_10_ pollution exposure in each Si-goon-gu in the 2015 winter

The National Institute of Environmental Research (NIER) measures the amount of air pollution per hour at 320 monitoring stations across the nation. The NIER CO and PM_10_ data from the winter of 2015 were extracted [28]. The data from the 38 monitoring stations that were located next to roads with heavy traffic (i.e., the road network monitoring stations) were excluded as these data are outliers. According to the address of each monitoring station, the remaining 282 monitoring stations were distributed variably across the 232 Si-goon-gu: 44 Si-goon-gu did not have monitoring stations and in some cases one Si-goon-gu had multiple monitoring stations. If there were multiple monitoring stations in a Si-goon-gu, the maximum moving average values were taken and the rest were deleted. To control for the impact of pollution from China, the data from the days when yellow dust was observed were excluded. The CO data in the dataset were expressed as the moving average of 8 hours while the PM_10_ data were expressed as the moving average of 24 h. Korean law stipulates that beyond the respective CO and PM_10_ thresholds of 9 ppm and 100 g/m^3^, the air quality is considered to be poor [29]. 73 Si-goon-gu were excluded in analysis because there are no monitoring station or missing value. Each Si-goon-gu has a number of days exceed PM_10_ and CO criteria. It assumed that the higher the number of days of baseline excess, the more severe the air pollution.

### Statistical analyses

Each item of the emission inventory, excluding the generation of electricity, was expressed as quartiles. Binomial negative analysis was performed to determine the dose-response relationship between the number of days in winter where the PM_10_ and CO levels were excessive and the population density, the number of car registrations/1000, the number of car accidents, the kwh of industrial power usage/km^2^, and whether electricity was generated locally or not. SAS software (version 9.4, SAS Institute Inc., Cary, NC, USA) was used for database management and the statistical analyses. Statistical significance was defined as *p* < .05.

## Results

Table 1 summarizes the descriptive statistics of each emission source. Statistics on each emission source and average number of winter days with excessive CO and PM_10_ levels by Si-Do scales are shown in Additional file 1 and in Additional file 2, respectively. Table 2 shows the average number of winter days with excessive CO and PM_10_ levels in the Si-goon-gu with low (Q1), moderate (Q2), high (Q3), and very high (Q4) population densities, numbers of car registrations, number of car accidents, and industrial power usage/km^2^. The average number of days the CO and PM_10_ levels exceeded the thresholds in the 90 and 142 Si-goon-gu that, respectively, did and did not generate electricity from fossil fuel power plants is also shown in Table 2.

**Table 1:** Descriptive statistics of the emission inventories of South Korea in the winter of 2015

	Mean	SD	Minimum (Min.)	Maximum (Max.)	Cut of level for quartiles		
					1st quartile	2nd quartile	3rd quartile
Population density (persons/km^2^)	4123	6431	20	27,938	98.27	459.19	6329.12
Number (No.) of car registrations (1000 cars)	72	74	4	232	16.43	48.90	98.36
No. of car accidents	1064	1078	2	5368	213.00	729.00	1521.00
Industrial power usage (Kwh/km^2^)	6,375,526	12,198,986	41,955	129,655,373	300,926.80	1,588,265.70	8,306,667.40
Generation of electricity (Kwh)	1000,841,589	2,274,966,981	93	10,673,238,998	1,309,273.00	52,356,385.00	820,165,148.00

**Table 2:** Number of days the particulate matter 10 (PM_10_) and carbon monoxide (CO) levels in the winter of 2015 exceeded government thresholds

	CO (days)			PM_10_ (days)		
	Mean ± SD	Min.	Max.	Mean ± SD	Min.	Max.
Population density (persons/km^2^)^a^						
Q1 (lowest)	8.3 ± 14.3	0	54	1.4 ± 1.2	0	4
Q2	28.4 ± 24.4	1	81	4.1 ± 4.7	0	23
Q3	41.8 ± 19.0	0	79	5.4 ± 5.3	0	21
Q4 (highest)	30.5 ± 21.1	0	66	2.2 ± 2.9	0	12
No. of car registrations^b^						
Q1 (lowest)	7.5 ± 13.9	0	54	2.0 ± 2.6	0	10
Q2	26.5 ± 24.1	0	77	3.0 ± 3.7	0	16
Q3	29.0 ± 19.3	0	72	2.9 ± 4.0	0	23
Q4 (highest)	43.2 ± 20.0	0	81	5.0 ± 5.2	0	21
No. of car accident^c^						
Q1 (lowest)	9.9 ± 14.6	0	54	2.1 ± 2.6	0	10
Q2	21.9 ± 23.0	0	77	2.1 ± 2.6	0	12
Q3	31.2 ± 19.6	0	72	3.6 ± 4.4	0	23
Q4 (highest)	43.0 ± 20.4	0	81	4.8 ± 5.2	0	21
Industrial power usage (Kwh/km^2^)^d^						
Q1 (lowest)	9.8 ± 13.8	0	46	1.6 ± 1.5	0	4
Q2	28.2 ± 26.5	0	81	4.2 ± 4.9	0	23
Q3	38.4 ± 18.6	1	78	5.0 ± 5.1	0	21
Q4 (highest)	33.8 ± 22.2	0	79	2.6 ± 3.7	0	16
Generation of electricity (Kwh)						
No	27.0 ± 22.8	0	77	2.3 ± 2.8	0	14
Yes	37.1 ± 21.2	0	81	5.1 ± 5.4	0	23

^a^population density(persons/km^2^): Q1 < 98.87, 98.87 ≤ Q2 < 459.19, 459.19 ≤ Q3 < 6329.12, 6329.12 ≤ Q4

^b^No. of car registrations(1000 cars): Q1 < 16.43, 16.43 ≤ Q2 < 48.90, 48.90 ≤ Q3 < 98.36, 98.36 ≤ Q4

^c^No. of car accident: Q1 < 213.00, 213.00 ≤ Q2 < 729.00, 729.00 ≤ Q3 < 1521.00, 1521.00 ≤ Q4

^d^Industrial power usage (Kwh/km^2^): Q1 < 300,926.80, 300,926.80 ≤ Q2 < 1,588,265.70, 1,588,265.70 ≤ Q3 < 8,306,667.40, 8,306,667.40 ≤ Q4

The number of days of excessive CO and PM_10_ increased steadily as the population density, and the industrial power usage/km^2^ increased. However, the peak number of days were observed in the 3Q, not 4Q. The number of days of excessive CO and PM_10_ increased steadily as the number of car accidents and the number of car registrations increased: the peak number of days were observed in the 4Q. The Si-goon-gu that did have power plants had more days of excessive CO and PM_10_ than the Si-goon-gu that did not.

Table 3 shows the results of the negative binomial regression analyses. Compared to the lowest population density (Q1), all three higher population densities associated significantly with higher CO and PM_10_ pollution. The association for both pollutants grew increasingly stronger as the population density rose from Q2 to Q3; thereafter (Q4), the association dropped somewhat. The same was observed for the industrial power usage: The numbers marked with an asterisk did not associate significantly with PM_10_ pollution compared to Q1. All of these findings are consistent with the trends shown in Table 2.

**Table 3:** Association between the number of days of excessive particulate matter 10 (PM_10_) and carbon monoxide (CO) in the winter of 2015 and concentration of putative contaminant sources

	CO		PM10	
	exp(β)	95% CI	exp(β)	95% CI
Population density (person/km^2^)^a^				
Q1(lowest)	1.00		1.00	
Q2	3.44	1.96–6.04	3.00	1.46–6.17
Q3	5.07	3.00–8.58	3.89	1.97–7.68
Q4(highest)	3.70	2.19–6.26	1.59*	0.79–3.18
No. of car registrations^b^				
Q1(lowest)	1.00		1.00	
Q2	3.53	1.96–6.38	1.50	0.70–3.21
Q3	3.86	2.22–6.72	1.45*	0.71–2.95
Q4(highest)	5.75	3.32–9.99	2.49	1.23–5.04
No. of car accident^c^				
Q1(lowest)	1.00		1.00	
Q2	2.20	1.23–3.93	1.00	0.47–2.13
Q3	3.14	1.84–5.36	1.67*	0.84–3.32
Q4(highest)	4.33	2.54–7.38	2.23	1.13–4.40
Industrial power usage (Kwh/ km^2^)^d^				
Q1(lowest)	1.00		1.00	
Q2	2.88	1.64–5.07	2.69	1.31–5.52
Q3	3.93	2.37–6.51	3.19	1.66–6.12
Q4(highest)	3.46	2.09–5.71	1.70*	0.88–3.27
Generation of electricity (Kwh)				
No	1.00		1.00	
Yes	1.37	1.01–1.86	2.27	1.60–3.21

^a^population density(persons/km^2^): Q1 < 98.87, 98.87 ≤ Q2 < 459.19, 459.19 ≤ Q3 < 6329.12, 6329.12 ≤ Q4

^b^No. of car registrations(car/1000): Q1 < 16.43, 16.43 ≤ Q2 < 48.90, 48.90 ≤ Q3 < 98.36, 98.36 ≤ Q4

^c^No. of car accident: Q1 < 213.00, 213.00 ≤ Q2 < 729.00, 729.00 ≤ Q3 < 1521.00, 1521.00 ≤ Q4

^d^Industrial power usage (Kwh/km^2^): Q1 < 300,926.80, 300,926.80 ≤ Q2 < 1,588,265.70, 1,588,265.70 ≤ Q3 < 8,306,667.40, 8,306,667.40 ≤ Q4

Compared to the lowest car accident counts (Q1), higher car accident counts associated significantly with higher CO and PM_10_ pollution (the only exception was the association between PM_10_ pollution and the Q3 car accident counts, which did not achieve statistical significance). There was a linear relationship between pollution and car mobile pollutants since the association steadily strengthened as the car accident counts and number of car registration rose. These findings are consistent with the trends shown in Table 2.

The Si-goon-gu that had power plants were significantly more likely to exceed both air pollution thresholds than the Si-goon-gu that lacked power plants.

## Discussion

Particulate matter and air pollution continue to be a problem during winter. There is a growing need to establish the cause in order to come up with countermeasures. The purpose of this study was also to identify the relationship between different variables, using official measurement data and other data provided by the country.

A key finding of this study was that there was a linear relationship between increasing mobile pollutant counts and increasing number of days of excessive CO and PM_10_ levels in Korea during the winter season of 2015. This is consistent with the fact that car emissions are a classic source of air pollutants such as CO, NO_x_, SO_x,_ and PM_10_. Indeed, a South Korean government report [1] showed that in 2013, car exhaust was responsible for 58.7% of all CO emissions and 10% of all PM_10_ emissions.

The present study found a consistent linear relationship between the number of car registrations and the days of excessive air pollution with CO and PM_10_: while the association did increase up to the 3Q, the association weakened in the 4Q. This is not consistent with a previous report that showed that air pollutant levels correlated significantly with several car-related statistics (major-road density, all-traffic density, and heavy-traffic density) [30]. Data from monitoring station where located roadside were excluded because the roadside’s outcome couldn’t represent all the area. Thus, our outcomes might underestimated the effect of motor vehicles.

We observed that the population density also showed a linear dose–response with air pollution until the 3Q: the highest population density associated less strongly with pollution than the smaller population densities. This is not consistent with a report from the United States of America that found a significant positive correlation between population density and air pollution [31]. However, the study conducted in the U.S only include major metropolitan areas where their population over one-million. Moreover, air pollution from population activities is not easy to explain. The results of each correlation analysis on population density and the number of car registration, number of car accidents, and industrial power usage were *r* = 0.31, 0.41 and 0.63 respectively. (Not presented as a separate table) Population density showed a weak correlation with mobile pollutants and a rather strong correlation with industrial power usage. Population density exhibited the same pattern as the negative regression analysis of industrial power usage. In other words, the 3Q group showed the greatest degree of contamination while the 4Q group showed a slight decrease in pollution. This suggests that while larger population translates to more industrial power usage, the content of industrial activity is deeply related to air pollution.

When we used industrial power usage as a measure of industrial activity, we found the same relationship with air pollution as population density: there was a linear dose–response relationship between industrial power usage and air pollution until the 3Q: the highest levels of industrial power usage associated less strongly with pollution than the 3rd levels of industrial power usage. We speculated that this may reflect differences in the specific type of industries that used industrial power. To address this, we assessed the types of industries in the Si-goon-gu that were in the 3Q and 4Q. Indeed, the industries in the 4Q were mainly service and healthcare businesses, which are unlikely to be air polluters. By contrast, the main industries in the 3Q manufactured electronic video and audio equipment, chemical products, and motor vehicles. Additional file 3 and Additional file 4 show the key industries in the third and 4Q of industrial power usage and how much industrial electricity they used in 2015 winter. Data from the KEPCO on the electricity usage by various industries in January, 2016, were used to compile this table [32].

Thus, the 3Q, but not 4Q of population density, and industrial power usage associated most strongly with air pollution. An analysis of the third and fourth Si-goon-gu showed that industrial electricity consumption was most concentrated in the downtown areas of Seoul and Busan, followed by the periphery of these metropolitan areas (Additional file 5). Additional file 6 showed that distribution of the Korea industrial complexes in 2015. Approximately 40% of the 3Q of industrial electricity consumption were concentrated in industrial complexes. These findings partially support the notion that PM_10_ and CO may be more affected by industrial complex activities than by population density.

Since there are relatively few fossil fuel power plants around the country, the Si-goon-gu with and without these plants were compared. The districts with these plants were significantly more likely to have air pollution than the districts that did not. This is consistent with a previous study [33].

The present study has several limitations. Since it had an ecological design, it was not possible to demonstrate a cause and effect relationship between the putative contaminant sources and air pollution. Moreover, all data used in the study were secondary data from the South Korean government. Third, while monitoring stations have been established all over the country to monitor air pollution levels, the stations are concentrated in metropolitan areas and around large cities. This shortcoming is difficult to overcome. While many studies use simulated pollution data, these data are merely estimates, and the purpose of this study was to examine the relationship between real air pollution data and putative sources. Another limitation is that due to substantial difficulties in estimating how much contaminants are produced by putative sources, we used surrogate variables to represent these sources. This limitation is partly overcome by the fact that we measured the population density, automobile density, and industrial activities in each district. Air pollution levels in a given country can be significantly affected by pollutants from surrounding countries. In South Korea, the air is often affected by pollution from China. However, we did not correct the measured air pollution levels for the direction of air flow, although we did exclude the data from the days when yellow dust was observed (19 days). Despite these efforts, external factors were not sufficiently excluded. Meanwhile, CO is a gaseous substance, so it is understood that the exposure in neighboring areas is a more appropriate material for explanation. Analyzing between pollutant and CO, not necessary to consider the effect from China. Finally, humidity, temperature and wind direction all influence air pollution, and the non-correction of these factors pose limitations in this study.

The present study showed the impact of major airborne sources on air pollution in South Korea in winter. Significantly, our study design allowed us to investigate whether all but one of the putative sources showed a positive dose–relationship with CO and PM_10_ pollution: all did. The remaining variable, power generation, which was expressed as a dichotomous variable, also associated with air pollution. In addition, we examined the contribution of motor vehicles to air pollution by not only assessing vehicle registration data but also the number of car accidents. In addition, we analyzed the effect of the type of industry in specific areas to explain why the third, not the fourth, highest industrial power usage associated most strongly with air pollution. Finally, the results are likely to be a good representation of the real situation because all data were from various parts of the government: this assures public confidence in the data.

## Conclusions

Air pollution is increasingly being seen as an important public health issue. To reduce air pollution, the incidence of exposure, and the impact on human health, it is important to identify the most significant sources of pollutants. This study showed that cars, industrial activity, fossil fuel plants, and population density associated significantly with CO and PM_10_ air pollution in South Korea in winter. It also showed that polluting industrial activity occurs on the periphery of large cities. Well-designed research studies that verify these findings are warranted.

## Additional files

Additional file 1: Descriptive statistics of the emission inventories of South Korea in the winter of 2015 by Si-Do. (DOCX 32 kb)

Additional file 2: Number of days the particulate matter 10 (PM_10_) and carbon monoxide (CO) levels in the winter of 2015 exceeded government thresholds by Si-Do. (DOCX 15 kb)

Additional file 3: Main types of industries in the districts that fell into the 3Q and 4Q of industrial power usage. (DOCX 15 kb)

Additional file 4: Mean amount of power sold by type of industries. (DOCX 15 kb)

Additional file 5: Distribution of the industrial power usage in South Korea in 2015 winter. The maps were generated by QGIS 2.18.6, which was provided as an open-source. (JPEG 711 kb)

Additional file 6: Distribution of the industrial complexes in South Korea [34]. (JPEG 586 kb)
