# Supplementary material for: Association between air pollution in the 2015 winter in South Korea and population size, car emissions, industrial activity, and fossil-fuel power plants: an ecological study
Source: Ann Occup Environ Med. 2018 Oct 5;30:60. doi: 10.1186/s40557-018-0273-5 (PMC6173887; doi:10.1186/s40557-018-0273-5)
Supplement: Supplementary file 1 — Descriptive statistics of the emission inventories of South Korea in the winter of 2015 by Si-Do. (DOCX 32 kb) [file 40557_2018_273_MOESM1_ESM.docx]

**Additional file 1. Descriptive statistics of the emission inventories of South Korea in the winter of 2015 by Si-Do**

|  |  | Mean | Standard Deviation | Maximum | Minimum |
| --- | --- | --- | --- | --- | --- |
| **Gangwon** | |  |  |  |  |
| Population density (persons/km^2^) | | 154 | 210 | 780 | 20 |
| No. of car registrations (1,000 cars) | | 29 | 32 | 115 | 7 |
| No. of car accidents | | 433 | 564 | 2,130 | 63 |
| Industrial power usage (Kwh/km^2^) | | 515,194 | 969,898 | 4,210,673 | 62,905 |
| Generation of electricity (Kwh) | | 321,680,104 | 350,275,408 | 743,904,051 | 931,815 |
| **Gyeonggi** | |  |  |  |  |
| Population density (persons/km^2^) | | 3,350 | 3,887 | 16,011 | 67 |
| No. of car registrations (1,000 cars) | | 127 | 101 | 385 | 14 |
| No. of car accidents | | 1,922 | 1,468 | 5,368 | 195 |
| Industrial power usage (Kwh/km^2^) | | 5,236,038 | 4,768,221 | 20,886,088 | 239,149 |
| Generation of electricity (Kwh) | | 783,566,592 | 938,710,907 | 3,640,390,874 | 809 |
| **Gyeongsangnam-do** | |  |  |  |  |
| Population density (persons/km^2^) | | 343 | 403 | 1,438 | 45 |
| No. of car registrations (1,000 cars) | | 68 | 106 | 445 | 8 |
| No. of car accidents | | 621 | 706 | 2,649 | 70 |
| Industrial power usage (Kwh/km^2^) | | 967,411 | 1,072,010 | 3,816,241 | 101,110 |
| Generation of electricity (Kwh) | | 1,685,195,716 | 3,362,147,555 | 8,410,216,856 | 294,545 |
| **Gyeongsangbuk-do** | |  |  |  |  |
| Population density (persons/km^2^) | | 158 | 183 | 683 | 22 |
| No. of car registrations (1,000 cars) | | 41 | 50 | 195 | 3 |
| No. of car accidents | | 518 | 748 | 2,675 | 2 |
| Industrial power usage (Kwh/km^2^) | | 671,639 | 973,430 | 3,957,286 | 41,955 |
| Generation of electricity (Kwh) | | 259,306,110 | 359,989,531 | 879,936,246 | 387,141 |
| **Gwangju** | |  |  |  |  |
| Population density (persons/km^2^) | | 3,530 | 1,876 | 6,495 | 1,782 |
| No. of car registrations (1,000 cars) | | 99 | 49 | 144 | 29 |
| No. of car accidents | | 1,905 | 921 | 2,687 | 706 |
| Industrial power usage (Kwh/km^2^) | | 4,785,460 | 2,283,228 | 8,696,985 | 3,115,424 |
| Generation of electricity (Kwh) | | 75,823,488 | 106,475,325 | 151,112,912 | 534,063 |
| **Daegu** | |  |  |  |  |
| Population density (persons/km^2^) | | 6,935 | 4,349 | 12,165 | 433 |
| No. of car registrations (1,000 cars) | | 113 | 63 | 211 | 48 |
| No. of car accidents | | 1,803 | 912 | 3,377 | 750 |
| Industrial power usage (Kwh/km^2^) | | 10,926,853 | 8,842,318 | 27,200,607 | 1,991,244 |
| Generation of electricity (Kwh) | | 250,355,735 | 370,725,176 | 677,565,669 | 13,173,015 |
| **Daejeon** | |  |  |  |  |
| Population density (persons/km^2^) | | 3,197 | 1,486 | 5,195 | 1,796 |
| No. of car registrations (1,000 cars) | | 104 | 45 | 168 | 68 |
| No. of car accidents | | 1,664 | 691 | 2,827 | 1,137 |
| Industrial power usage (Kwh/km^2^) | | 5,200,984 | 3,118,515 | 10,326,815 | 1,905,112 |
| Generation of electricity (Kwh) | | 49,737,809 | 23,781,555 | 66,553,907 | 32,921,710 |
| **Busan** | |  |  |  |  |
| Population density (persons/km^2^) | | 9,688 | 5,438 | 17,488 | 439 |
| No. of car registrations (1,000 cars) | | 63 | 34 | 135 | 13 |
| No. of car accidents | | 989 | 434 | 1,782 | 320 |
| Industrial power usage (Kwh/km^2^) | | 12,829,340 | 8,606,504 | 39,607,215 | 1,297,260 |
| Generation of electricity (Kwh) | | 418,688,738 | 811,210,300 | 1,635,183,928 | 93 |
| **Seoul** | |  |  |  |  |
| Population density (persons/km^2^) | | 17,488 | 4,922 | 27,938 | 6,565 |
| No. of car registrations (1,000 cars) | | 102 | 43 | 216 | 40 |
| No. of car accidents | | 1,853 | 966 | 5,357 | 924 |
| Industrial power usage (Kwh/km^2^) | | 21,671,186 | 10,949,174 | 64,266,833 | 10,374,840 |
| Generation of electricity (Kwh) | | 64,219,113 | 75,423,176 | 214,286,649 | 9,144,784 |
| **Sejong^*^** | |  |  |  |  |
| Population density (persons/km^2^) | | 336 |  |  |  |
| No. of car registrations (1,000 cars) | | 76 |  |  |  |
| No. of car accidents | | 442 |  |  |  |
| Industrial power usage (Kwh/km^2^) | | 1,504,342 |  |  |  |
| Generation of electricity (Kwh) | | 961,785,140 |  |  |  |
| **Ulsan** | |  |  |  |  |
| Population density (persons/km^2^) | | 3,520 | 2,642 | 6,453 | 283 |
| No. of car registrations (1,000 cars) | | 87 | 27 | 129 | 56 |
| No. of car accidents | | 1,265 | 635 | 2,379 | 777 |
| Industrial power usage (Kwh/km^2^) | | 15,185,233 | 20,226,782 | 50,762,907 | 3,506,212 |
| Generation of electricity (Kwh) | | 1,171,684,461 | 1,656,984,126 | 2,343,349,172 | 19,749 |
| **Incheon** | |  |  |  |  |
| Population density (persons/km^2^) | | 7,226 | 6,251 | 17,404 | 120 |
| No. of car registrations (1,000 cars) | | 111 | 81 | 218 | 7 |
| No. of car accidents | | 1,377 | 1,031 | 2,992 | 36 |
| Industrial power usage (Kwh/km^2^) | | 22,558,961 | 38,484,176 | 129,655,373 | 321,547 |
| Generation of electricity (Kwh) | | 2,618,968,317 | 4,348,064,881 | 10,673,238,998 | 2,335,911 |
| **Jeollanam-do** | |  |  |  |  |
| Population density (persons/km^2^) | | 342 | 967 | 4,636 | 56 |
| No. of car registrations (1,000 cars) | | 28 | 26 | 93 | 8 |
| No. of car accidents | | 370 | 437 | 1,618 | 67 |
| Industrial power usage (Kwh/km^2^) | | 939,140 | 1,783,925 | 6,826,105 | 105,923 |
| Generation of electricity (Kwh) | | 493,341,122 | 872,120,186 | 2,269,070,235 | 85,946 |
| **Jeollabuk-do** | |  |  |  |  |
| Population density (persons/km^2^) | | 392 | 828 | 3,177 | 34 |
| No. of car registrations (1,000 cars) | | 44 | 63 | 236 | 6 |
| No. of car accidents | | 654 | 991 | 3,680 | 59 |
| Industrial power usage (Kwh/km^2^) | | 1,089,304 | 1,852,809 | 5,605,791 | 69,170 |
| Generation of electricity (Kwh) | | 340,641,252 | 717,428,665 | 1,623,422,362 | 3,855 |
| **Jeju** | |  |  |  |  |
| Population density (persons/km^2^) | | 320 | 196 | 459 | 182 |
| No. of car registrations (1,000 cars) | | 170 | 161 | 283 | 56 |
| No. of car accidents | | 1,529 | 1,165 | 2,353 | 705 |
| Industrial power usage (Kwh/km^2^) | | 653,288 | 115,697 | 735,098 | 571,478 |
| Generation of electricity (Kwh) | | 208,498,096 | 196,723,344 | 347,602,506 | 69,393,685 |
| **Chungcheongnam-do** | |  |  |  |  |
| Population density (persons/km^2^) | | 271 | 248 | 941 | 67 |
| No. of car registrations (1,000 cars) | | 48 | 54 | 219 | 9 |
| No. of car accidents | | 712 | 976 | 3,968 | 120 |
| Industrial power usage (Kwh/km^2^) | | 1,415,522 | 1,657,252 | 5,702,552 | 177,613 |
| Generation of electricity (Kwh) | | 4,650,437,284 | 4,869,350,166 | 10,015,674,187 | 1,309,273 |
| **Chungcheongbuk-do** | |  |  |  |  |
| Population density (persons/km^2^) | | 211 | 249 | 884 | 40 |
| No. of car registrations (1,000 cars) | | 50 | 84 | 295 | 9 |
| No. of car accidents | | 658 | 1,033 | 3,632 | 110 |
| Industrial power usage (Kwh/km^2^) | | 879,706 | 870,538 | 2,955,151 | 146,522 |
| Generation of electricity (Kwh) | | 54,871,913 | 42,752,636 | 85,102,592 | 24,641,234 |
| *Sejong has no si-goon-gu division. | | | | | |
