# Supplementary material for: Association between air pollution in the 2015 winter in South Korea and population size, car emissions, industrial activity, and fossil-fuel power plants: an ecological study
Source: Ann Occup Environ Med. 2018 Oct 5;30:60. doi: 10.1186/s40557-018-0273-5 (PMC6173887; doi:10.1186/s40557-018-0273-5)
Supplement: Supplementary file 2 — Number of days the particulate matter 10 (PM10) and carbon monoxide (CO) levels in the winter of 2015 exceeded government thresholds by Si-Do. (DOCX 15 kb) [file 40557_2018_273_MOESM2_ESM.docx]

**Additional file 2.** **Number of days the particulate matter 10 (PM_10_) and carbon monoxide (CO) levels in the winter of 2015 exceeded government thresholds by Si-Do**

|  | CO(days) | | | PM_10_(days) | | |
| --- | --- | --- | --- | --- | --- | --- |
|  | mean±sd | Max. | Min. | mean±sd | Max. | Min. |
| Gangwon | 27.1±30.2 | 81 | 0 | 2.9±4.6 | 14 | 0 |
| Gyeonggi | 42.7±18.7 | 70 | 5 | 7.7±6.1 | 23 | 1 |
| Gyeongsangnam-do | 18.9±16.6 | 44 | 0 | 2.6±2.2 | 7 | 1 |
| Gyeongsangbuk-do | 30.8±29.5 | 78 | 2 | 2.2±1.4 | 4 | 0 |
| Gwangju | 27.0±13.9 | 46 | 9 | 1.0±1.7 | 4 | 0 |
| Daegu | 21.8±13.2 | 38 | 2 | 3.8±4.0 | 10 | 0 |
| Daejeon | 44.4±11.6 | 56 | 25 | 2.2±3.8 | 9 | 0 |
| Busan | 3.5±8.6 | 35 | 0 | 2.4±2.5 | 8 | 0 |
| Seoul | 37.8±13.4 | 60 | 12 | 1.0±1.1 | 4 | 0 |
| Sejong^*^ | - | - | - | - | - | - |
| Ulsan | 35.2±25.6 | 79 | 13 | 1.8±0.8 | 3 | 1 |
| Incheon | 46.6±19.4 | 67 | 10 | 5.9±5.8 | 16 | 0 |
| Jeollanam-do | 28.7±20.8 | 58 | 2 | 1.0±1.3 | 3 | 0 |
| Jeollabuk-do | 24.0±19.1 | 51 | 0 | 3.9±1.8 | 6 | 1 |
| Jeju | 3.0±2.8 | 5 | 1 | 3.5±3.5 | 6 | 1 |
| Chungcheongnam-do | 38.0±28.7 | 68 | 4 | 2.8±2.9 | 7 | 0 |
| Chungcheongbuk-do | 49.2±27.0 | 70 | 2 | 5.6±5.5 | 15 | 2 |
| *Sejong has no air pollution monitoring stations | | | | | | |
