# Supplementary material for: Association between air pollution in the 2015 winter in South Korea and population size, car emissions, industrial activity, and fossil-fuel power plants: an ecological study
Source: Ann Occup Environ Med. 2018 Oct 5;30:60. doi: 10.1186/s40557-018-0273-5 (PMC6173887; doi:10.1186/s40557-018-0273-5)
Supplement: Supplementary file 3 — Main types of industries in the districts that fell into the 3Q and 4Q of industrial power usage. (DOCX 15 kb) [file 40557_2018_273_MOESM3_ESM.docx]

**Additional file 3. Main types of industries in the districts that fell into the 3^rd^ and 4^th^ quartiles of industrial power usage**

| 3rd quartile | | | |  | 4th quartile | | | |
| --- | --- | --- | --- | --- | --- | --- | --- | --- |
| Type of industry | Industrial power usage (Mwh) | % | cumulative % |  | Type of industry | Industrial power usage (Mwh) | % | cumulative % |
| Manufacture of electronic video and audio equipment | 3332.60 | 16.73 | 16.73 |  | Simple services | 3796.66 | 35.62 | 35.62 |
| Simple services | 3217.49 | 16.16 | 32.89 |  | for home | 2163.08 | 20.29 | 55.91 |
| Manufacture of basic metals | 3082.17 | 15.48 | 48.37 |  | Manufacture of chemical products | 815.60 | 7.65 | 63.56 |
| Manufacture of chemical products | 2012.48 | 10.11 | 58.47 |  | Manufacture of basic metals | 391.79 | 3.68 | 67.23 |
| For home | 1909.11 | 9.59 | 68.06 |  | Manufacture of refined petroleum products | 355.41 | 3.33 | 70.57 |
| Manufacture of motor vehicles | 901.68 | 4.53 | 72.59 |  | Manufacture of electronic video and audio equipment | 296.49 | 2.78 | 73.35 |
| Manufacture of refined petroleum products | 618.51 | 3.11 | 75.70 |  | Public health centers | 271.62 | 2.55 | 75.90 |
| other machinery | 427.58 | 2.15 | 77.84 |  | other public purposes | 263.97 | 2.48 | 78.37 |
| Manufacture of textiles, except apparel | 386.60 | 1.94 | 79.78 |  | public office | 243.42 | 2.28 | 80.66 |
| Manufacture of pulp, paper and paper products | 386.60 | 1.94 | 81.73 |  | other machinery | 219.18 | 2.06 | 82.71 |
