# Supplementary material for: Association between air pollution in the 2015 winter in South Korea and population size, car emissions, industrial activity, and fossil-fuel power plants: an ecological study
Source: Ann Occup Environ Med. 2018 Oct 5;30:60. doi: 10.1186/s40557-018-0273-5 (PMC6173887; doi:10.1186/s40557-018-0273-5)
Supplement: Supplementary file 4 — Mean amount of power sold by type of industries. (DOCX 15 kb) [file 40557_2018_273_MOESM4_ESM.docx]

**Additional file 4. Mean amount of power sold by type of industries**

|  | 3rd quartile | | 4th quartile | |  |
| --- | --- | --- | --- | --- | --- |
| Type of industry | Mean(Mwh) | SD | Mean(Mwh) | SD | p-value* |
| Manufacture of electronic video and audio equipment | 60.59 | 149.03 | 5.29 | 16.67 | 0.01 |
| Simple services | 56.45 | 40.01 | 65.46 | 48.76 | 0.28 |
| Manufacture of basic metals | 55.04 | 157.15 | 7.12 | 19.73 | 0.03 |
| Manufacture of chemical products | 35.94 | 128.12 | 14.06 | 81.50 | 0.28 |
| For home | 33.49 | 22.53 | 37.29 | 21.61 | 0.36 |
| Manufacture of motor vehicles | 15.82 | 26.94 | 3.76 | 8.25 | 0.00 |
| Manufacture of refined petroleum products | 15.86 | 57.84 | 17.77 | 73.56 | 0.91 |
| other machinery | 7.50 | 15.35 | 3.78 | 7.68 | 0.10 |
| Manufacture of textiles, except apparel | 7.03 | 19.64 | 3.67 | 12.21 | 0.28 |
| Manufacture of pulp, paper and paper products | 6.90 | 15.96 | 2.11 | 8.26 | 0.05 |
| Public health centers | 3.32 | 8.34 | 4.68 | 26.21 | 0.71 |
| other public purposes | 5.10 | 5.80 | 4.55 | 4.21 | 0.56 |
| public office | 4.70 | 4.45 | 4.20 | 4.72 | 0.56 |

**T-test was conducted to compare amount of sold electricity in 3^rd^ and 4^th^ quartile groups.*
