# Supplementary figures and images for: Association between air pollution in the 2015 winter in South Korea and population size, car emissions, industrial activity, and fossil-fuel power plants: an ecological study
Source: Ann Occup Environ Med. 2018 Oct 5;30:60. doi: 10.1186/s40557-018-0273-5 (PMC6173887; doi:10.1186/s40557-018-0273-5)

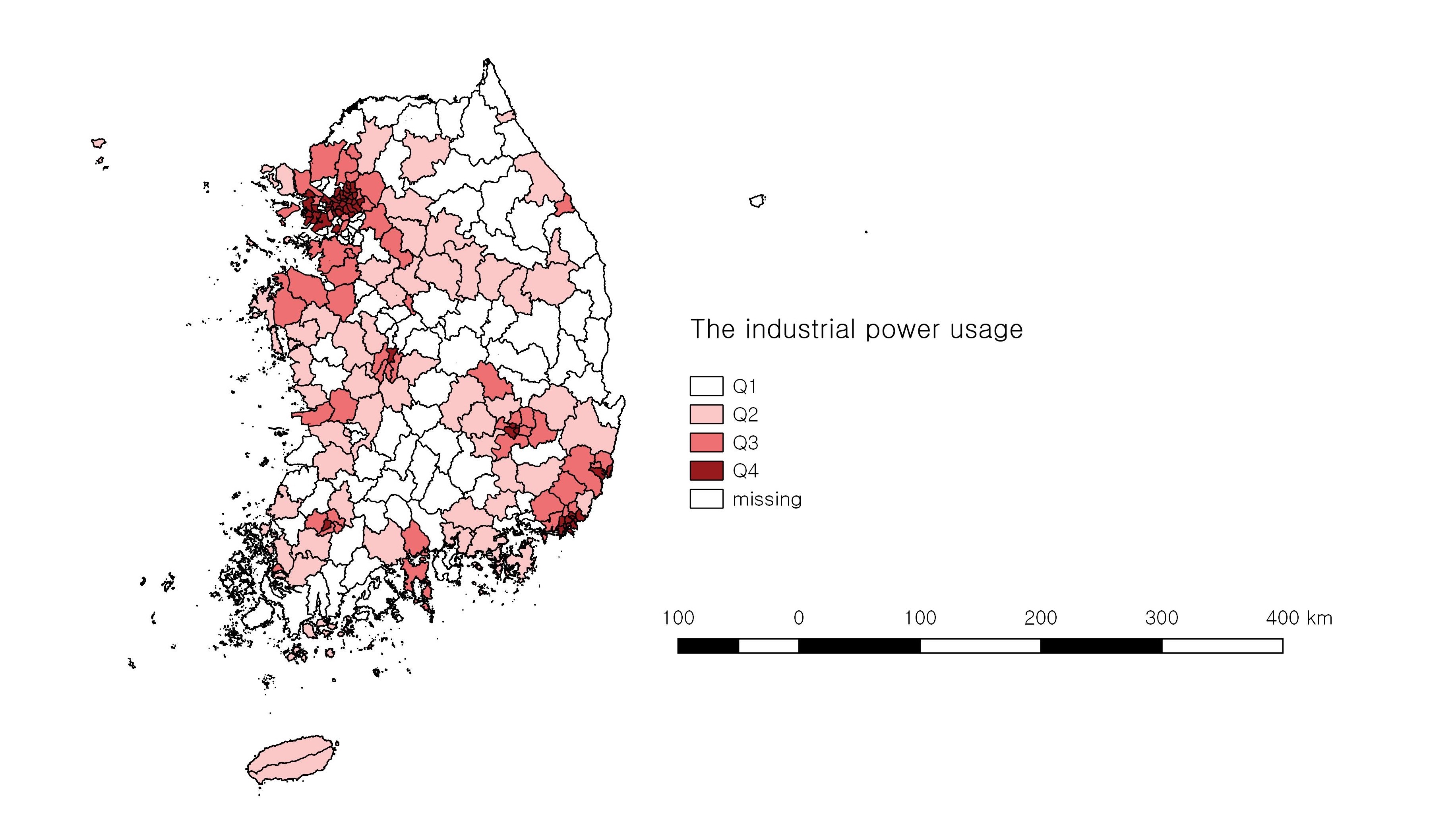

Supplement: Supplementary file 5 — Distribution of the industrial power usage in South Korea in 2015 winter. The maps were generated by QGIS 2.18.6, which was provided as an open-source. (JPEG 711 kb) [file 40557_2018_273_MOESM5_ESM.jpeg]

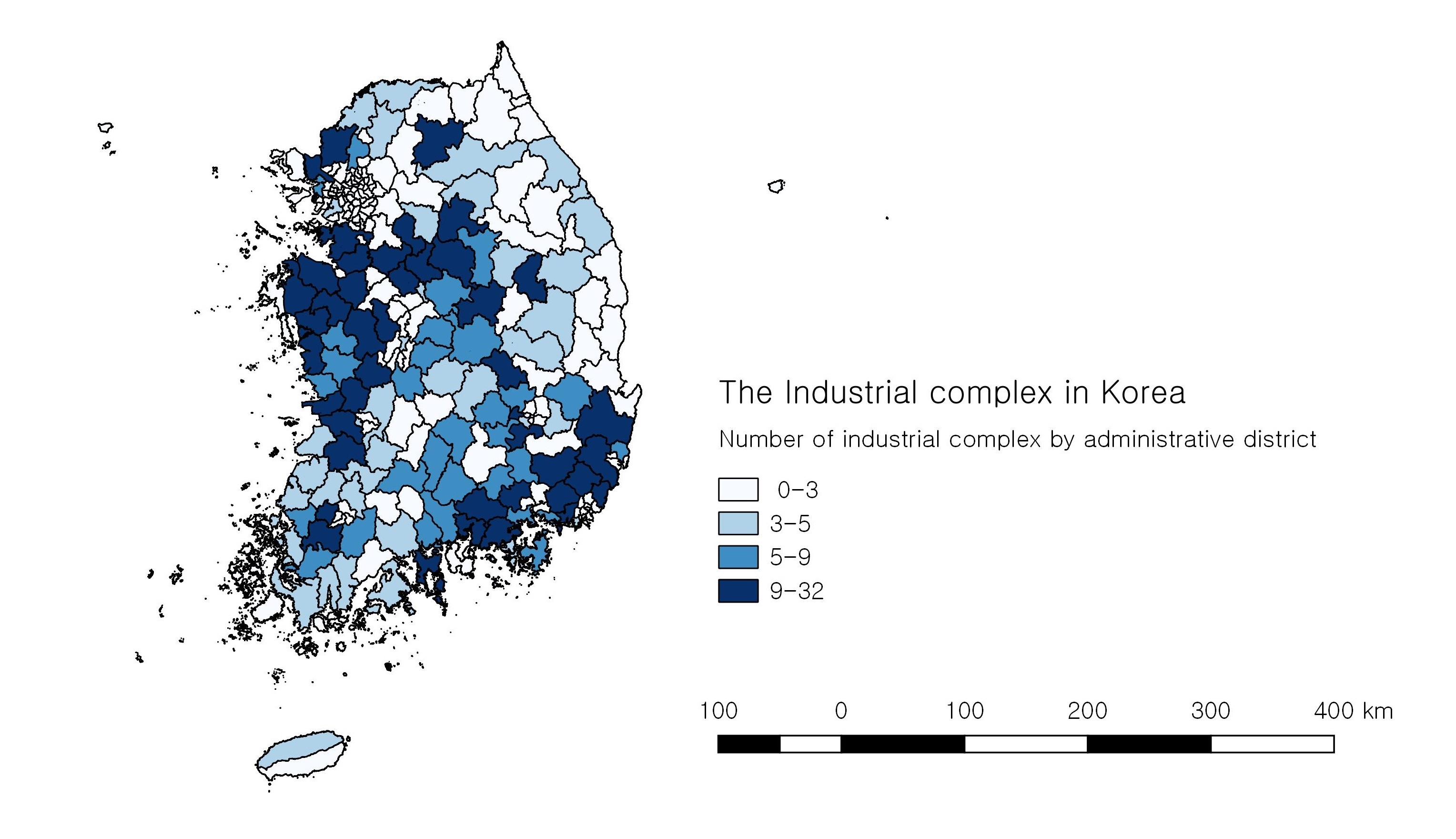

Supplement: Supplementary file 6 — Distribution of the industrial complexes in South Korea [34]. (JPEG 586 kb) [file 40557_2018_273_MOESM6_ESM.jpeg]
